# Editorial: Insect behavioral adaptations and immune responses to stress

**DOI:** 10.3389/fphys.2023.1244589

**Published:** 2023-07-04

**Authors:** Ioannis Eleftherianos, Amr A. Mohamed, Gianluca Tettamanti, Wei Zhang

**Affiliations:** ^1^ Department of Biological Sciences, Infection and Innate Immunity Laboratory, The George Washington University, Washington, DC, United States; ^2^ Department of Entomology, Faculty of Science, Cairo University, Giza, Egypt; ^3^ Department of Biotechnology and Life Sciences, University of Insubria, Varese, Italy; ^4^ National Key Laboratory of Green Pesticide, Key Laboratory of Green Pesticide and Agricultural Bioengineering, Ministry of Education, Guizhou University, Guiyang, China

**Keywords:** sensation, signal processing, adaptation, stress factors, pathogens, innate immunity, behavioral response

Insects are among the planet’s most successful animals. They exist in almost all ecosystems and are therefore exposed to a variety of threats, including extreme weather and climate-related events, nutritional deprivation, pesticide application, pathogen infection, and intra- or inter-species competition (Stork, 2018). Behavioral adaptations and innate immune capacity are one of the most effective and timely adaptation strategies for insects to withstand biotic and abiotic stressors and successfully persist in the environment through a series of regulatory processes (de Roode and Lefèvre, 2012; Zhang et al., 2019). These behavioral and immune adaptations of insects to the various stressors promote their spread, propagation, and evolution, and some insect species have even emerged as important agricultural pests or dangerous disease-spreading vectors (Chown and Terblanche, 2006; Garnas, 2018).

The aim of this Research Topic was to develop a platform to share the latest progress on insect behavioral and innate immune adaptations to biotic and abiotic stresses. Unraveling the regulatory processes of these adaptive responses from the genetic, physiological, and ecological levels is helpful for understanding how insects interact with the environment, hosts and pathogens (Coolen et al., 2022). These studies also provide insights into improving the biocontrol of agricultural insect pests and vectors of human diseases. In addition, this information contributes to expanding the use of insects as *in vivo* models for studying the dynamics of ecological evolution and host-pathogen interactions (Scully and Bidochka, 2006; Paez and Fleming-Davies, 2020).

The four published articles in this Research Topic focus on the role of insect olfactory genes, odorant-degrading enzymes, chemosensory proteins, and odorant receptors in host recognition. The reported research spans three insect orders (Thysanoptera, Hemiptera, and Coleoptera) and includes important pests and disease vectors. Understanding the molecular and functional bases of the insect olfactory system and how it interacts and coordinates with insect physiology to alter behavior is crucial for devising integrated pest management strategies.

Li et al. performed a comprehensive transcriptomic analysis through RNA-sequencing to determine the molecular mechanisms of the olfactory response in different developmental stages of the bean flower thrip, *Megalurothrips usitatus* (Li et al.). Bioinformatic analysis identified a large set of differentially expressed genes including one odorant-binding protein (MusiOBP1) and one chemosensory protein (MusiCSP1). Obtaining the full-length sequence of MusiOBP1 and MusiCSP1 facilitated their protein expression analysis and purification. Fluorescence binding assays further showed that MusiOBP1 has a strong binding affinity for β-citronellol (a natural acyclic monoterpenoid) while MusiCSP1 strongly binds to 3-hydroxy-2-methyl-4-pvrone (commonly known as maltol; a naturally occurring organic compound that is used primarily as a flavor enhancer). These are important findings with potentially translational applications through developing new behavioral pest control procedures.

Kuang et al. investigated antennae-abundant odorant-degrading enzymes in the Asian citrus psyllid, *Diaphorina citri* (Kuang et al.). They used a deep sequencing of RNA libraries approach to isolate cytochrome P450 genes from the antennae and body of male and female *D. citri*. Further qRT-PCR analyses showed that seven antennae-enriched DcCYP genes were antenna-biased and consistently expressed from the first instar to the female and male adult stage. One exception was gene *DcCYP6j1*, which was expressed at higher levels in male than in the female antennae. These findings will assist in elucidating the *D. citri* odorant recognition mechanism that will in turn lead to the development of sustainable tactics to restrict the spread of this insect vector.

Chen et al. examined the function of odorant receptor genes in the beetle *Diorhabda tarsalis* (Chen et al.). The identification of odorant receptor genes by PacBio RS II platform was followed by phylogenetic analysis, which showed that the odorant coreceptor gene *DtarORco* was mainly expressed in the antenna of female and male beetles. Successful silencing of *DtarORco* through RNA interference resulted in reduced electrophysiological responses to host localization odor signals in *D. tarsalis* which was accompanied by significant impairment of the beetles to detect hosts. This information reveals the key role of *DtarORco* in behavior and host location, a significant piece of information, that is, required for establishing environmentally friendly control practices for this insect pest.

Zhang et al. explored chemoreception in the tea green leafhopper, *E. onukii*, by focusing on three chemosensory proteins (Zhang et al.). *EonuCSP4*, *EonuCSP 6-1*, and *EonuCSP6-2* were expressed at high levels in the antennae of *E. onukii* and fluorescence binding assays together with behavioral tests indicated that *EonuCSP 6-1* had relatively high binding affinity and preference for benzaldehyde. These findings provide strong evidence for the chemoreception properties of *EonuCSP 6-1*, which may open new avenues for preventing the food-orientation behavior of this pest via obstructing the detection of chemosensory cues.

The published papers in this Research Topic present novel information on the functional basis of behavioral adaptations and innate immune responses in various insect species. This knowledge is essential because it contributes to a better understanding of the mechanisms that regulate these fundamental biological adaptation processes in insects (Lacey et al., 2015). In addition, findings from these works can be used in the future to develop novel strategies to fight against noxious insects that impact agriculture and human life (Rupawate et al., 2023). Viewed through a lens of evolutionary timescales, insect behavioral and immune adaptations may be exquisite to the point of being apparently paradoxical observations. This means that although insect behaviors are innate, they can be environmentally and physiologically adapted. Similarly, the insect immune response to infection differs according to the type of pathogen and infection route, the type of host and the presence of microbiota and its microbial composition (Westwick and Rittschof, 2021; Bruno et al., 2022; Eleftherianos et al., 2022). Finally, we would like to thank all the authors, reviewers, and editors for their contributions to this article Research Topic.
